# ﻿Triploidy in parthenogenetic Chinese *Helophorus aquila*Angus et al., 2014

**DOI:** 10.3897/compcytogen.19.169353

**Published:** 2025-10-07

**Authors:** Robert B. Angus, Fenglong Jia

**Affiliations:** 1 Institute of Entomology, School of Life Sciences, Sun Yat-sen University, Guangzhou, 510275, Guangdong, China Sun Yat-sen University Guangdong China; 2 Department of Life Sciences (Insects), The Natural History Museum, Cromwell Road, London SW7 5BD, UK Department of Life Sciences (Insects), The Natural History Museum London United Kingdom

**Keywords:** China, chromosomes, *Helophorus aquila*, triploid

## Abstract

Checking old unphotographed slides of chromosome preparations in the possession of R.B.A. revealed one slide labelled “*frater* ♀7g 6/6/13 ✓”. The beetle with these data is a female paratype of *H. aquila* Angus et al., in the general collection of the Natural History Museum, London. One almost complete dividing nucleus was found, with 32 chromosomes, indicating a triploid nucleus with one chromosome lost in the course of preparation of the slide.

## ﻿Introduction

Angus et al. (2014) noted that the *A. aquila* material was originally thought to belong to *H. frater* d’Orchymont, 1926 and only recognised as a separate species following discovery of males with a distinctive aedeagus. Angus et al. noted that only three male *H. aquila* were found out of 102 specimens listed as the type series and in the key to species (p.217) described the species as “parthenogenetic to some extent, males absent or very rare”, contrasted with *H. frater* “clearly bisexual species with males and females present in approximately equal numbers”.

The discovery of the nucleus reported here shows *H. aquila* to be a third species, along with *H. brevipalpis* Bedel, 1881 and *H. orientalis* Motschulsky, 1860 to include triploid parthenogenetic females (Angus 1992; Angus, Jia 2020).

## ﻿Material and methods

The material studied comprises one slide with the label “*frater* ♀7g 6/6/13 ✓”, originally prepared in 2013. ♀7 is the specimen number, g is gut and ✓ means R.B.A. thought there was useful material present. The details of preparation are as described by Angus (2025). No C-banding could be attempted as when the slide was cleaned by immersion first in xylene, then in absolute ethanol, the triploid nucleus could no longer be found, although most other material was still present. Loss of a nucleus from the slide is an unavoidable hazard with this procedure.

## ﻿Results

**Figure 1.**
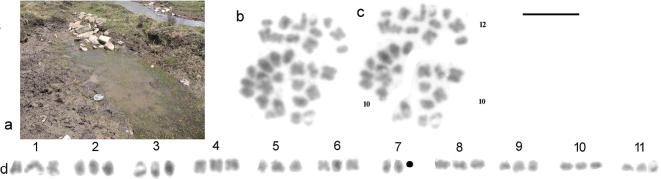
**a** collection site for the triploid female *H. aquila***b, c** the triploid nucleus **b** as found **c** with sections separated for ease of counting **d** the chromosomes arranged as a karyogram. The missing chromosome is indicated by a bold dot. Scale bar: 5 μm.

The collection site is shown in Fig. 1a. The principal target of collection at this site *Boreonectes emmerichi* (Falkenström, 1936) (Angus et al. 2015) and a small sieve was used for collecting. The triploid nucleus is shown in Fig. 1b (as found), c (with sections slightly separated for ease of counting). Fig. 1d shows the chromosomes arranged as a karyogram. The existence of triploid sets is very clear.

## ﻿Conclusion

*Helophorus aquila* is shown to include triploid parthenogenetic females.
